# Bioluminescence imaging of G protein-coupled receptor activation in living mice

**DOI:** 10.1038/s41467-017-01340-7

**Published:** 2017-10-27

**Authors:** Mari Kono, Elizabeth G. Conlon, Samantha Y. Lux, Keisuke Yanagida, Timothy Hla, Richard L. Proia

**Affiliations:** 1 Genetics of Development and Disease Branch, National Institute of Diabetes and Digestive and Kidney Diseases, NIH, Bethesda, MD 20892 USA; 2 Vascular Biology Program, Boston Children’s Hospital, Department of Surgery, Harvard Medical School, Boston, MA 02115 USA

## Abstract

G protein-coupled receptors (GPCRs), a superfamily of cell-surface receptors involved in virtually all physiological processes, are the major target class for approved drugs. Imaging GPCR activation in real time in living animals would provide a powerful way to study their role in biology and disease. Here, we describe a mouse model that enables the bioluminescent detection of GPCR activation in real time by utilizing the clinically important GPCR, sphingosine-1-phosphate receptor 1 (S1P_1_). A synthetic S1P_1_ signaling pathway, designed to report the interaction between S1P_1_ and β-arrestin2 via the firefly split luciferase fragment complementation system, is genetically encoded in these mice. Upon receptor activation and subsequent β-arrestin2 recruitment, an active luciferase enzyme complex is produced, which can be detected by in vivo bioluminescence imaging. This imaging strategy reveals the dynamics and spatial specificity of S1P_1_ activation in normal and pathophysiologic contexts in vivo and can be applied to other GPCRs.

## Abstract (web summary)

G protein-coupled receptors are involved in numerous physiological functions, thus, they represent potential pharmaceutical targets. Here Kono et al. describe a new mouse model to image GPCR activation in real-time by exploiting firefly split luciferase fragment complementation that can be detected by bioluminescence imaging.

## Introduction

G protein-coupled receptors (GPCRs) (also known as seven-transmembrane-domain receptors) are the largest and most diverse gene superfamily in the human genome, comprising greater than 3% of the protein coding genes^1–3^. GPCRs are widely expressed, initiate cellular signal transduction by a diverse array of extracellular ligands, and are involved in virtually all physiological functions^1,4^. They are also extremely important in clinical medicine as a major drug target class. It is estimated that ~30% of approved pharmaceutical drugs are directed to GPCRs^4,5^. Despite their enormous biological and clinical importance, technologies to image GPCR activation in their native physiologic environment in real time are lacking, hampering a full understanding of the spatiotemporal dynamics of their activation in vivo by endogenous or exogenous ligands.

Sphingosine-1-phosphate receptor 1 (S1P_1_) is one of a five-member GPCR family with high affinity for the bioactive sphingolipid, sphingosine-1-phosphate (S1P). It is ubiquitously expressed among tissues and is highly enriched in endothelial cells, where it serves as a key regulator of vascular barrier function^6–8^. S1P_1_ also performs important actions in the immune and nervous systems^7^. Its endogenous sphingolipid ligand, S1P, is produced by all cells and is carried by lipoproteins and albumin in the circulation^7^. S1P–S1P_1_ signaling has been linked to diverse disease processes, including infection, multiple sclerosis, atherosclerosis, inflammatory bowel disease, and cancer^6,7,9^. FTY720 (fingolimod/Gilenya^TM^), an S1P_1_ ligand, is an FDA-approved drug for the treatment of remitting-relapsing multiple sclerosis^10^.

Ligand-activated GPCRs stimulate intracellular signaling pathways via G proteins^11,12^ and other effectors, including β-arrestins^13–16^. β-Arrestin binding to activated GPCRs also mediates receptor desensitization, internalization, and recycling. The near-universal interaction between GPCRs and β-arrestins following agonist binding has been employed in assays for quantifying receptor activation^17–21^. We recently developed a mouse model for the detection of activated S1P_1_ in which a green fluorescent protein (GFP) reporter gene was activated^22^. This model enabled the identification of cells in which receptor activation has occurred in a cumulative manner, but it is not suited for intravital imaging of real-time activation of GPCR signaling.

Here, we describe a genetic model in mice that enables the imaging of S1P_1_ signaling in real time in the native physiological environment of the receptor. These S1P_1_ signaling mice were genetically designed to report the interaction between S1P_1_ and β-arrestin2 upon receptor activation via firefly split luciferase fragment complementation, which produces an active enzyme complex whose activity can be detected by bioluminescence imaging. The S1P_1_ signaling mice respond to synthetic ligands in accordance to the specificity of the native receptor and the stability of pharmacologic agonists. The S1P_1_ signaling mice also reveal the timing and anatomical localization of receptor activation by the endogenously synthesized S1P ligand during endotoxin-induced systemic inflammation. This paradigm can be applied to other members of the clinically important GPCR family to enable the study of receptor activation in their in vivo settings.

## Results

### Generation of S1P_1_ luciferase signaling mice

**Fig. 1:**
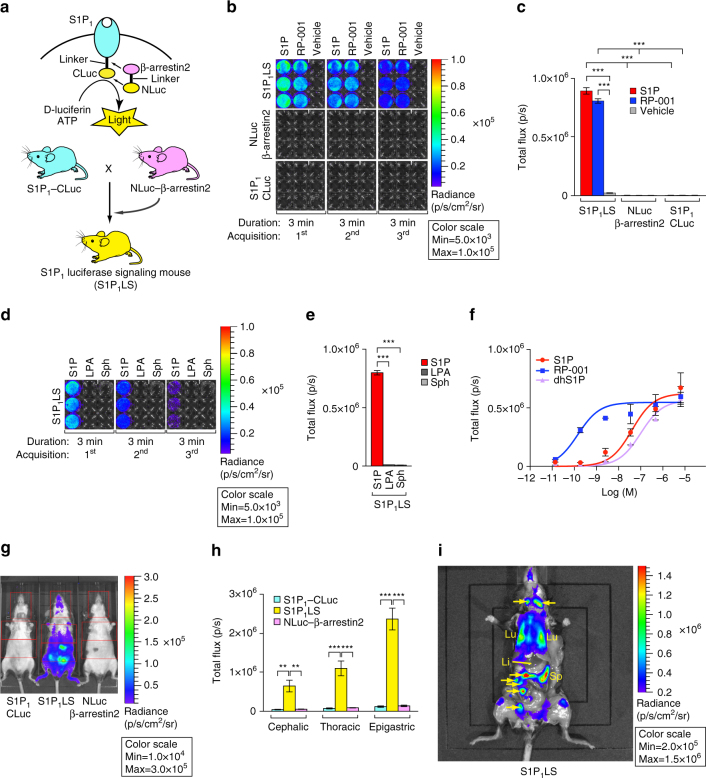
Generation of S1P_1_ luciferase signaling mice and detection of S1P_1_ activation. **a** (top), Schematic of the firefly split luciferase complementation design to monitor S1P_1_–β-arrestin2 interactions. **a** (bottom), Mouse mating scheme to derive the S1P_1_ luciferase signaling (S1P_1_LS) mice. **b**–**f** Activation of S1P_1_ in MEFs. **b** S1P_1_ luciferase signaling (S1P_1_LS) MEFs heterozygous for the S1P_1_–CLuc allele and homozygous for the NLuc–β-arrestin2 allele, MEFs heterozygous for the S1P_1_–CLuc allele, and MEFs homozygous for the NLuc–β-arrestin2 allele were treated with S1P, RP-001 (10^−6^ M), or vehicle in triplicate, and immediately subjected to BLI. Intensities are shown in units of photons/sec/cm^2^/steradian; p/sec/cm^2^/sr. **c** The bioluminescence from three sequential 3-min acquisitions in **b** was averaged. Data represent the mean ± SEM. *n* = 3 for each group. **d** MEFs from S1P_1_ luciferase signaling (S1P_1_LS) mice were exposed to S1P, sphingosine (Sph), or LPA (10^−6^ M) in triplicate, and immediately subjected to BLI. Imaging was for sequential 3-min periods. **e** The bioluminescence from three sequential 3-min acquisitions in **d** was averaged. Data represent the mean ± SEM, *n* = 3 for each group. Experiments in **b** and **d** were repeated twice. **f** Dose–response of S1P_1_ activation in S1P_1_ luciferase signaling MEFs. MEFs derived from S1P_1_ luciferase signaling mice, then exposed in triplicate to various concentrations of S1P, RP-001, or dhS1P and immediately subjected to BLI for 30 min. The experiment was repeated twice. Data represent the mean ± SEM. **g**, **h** Basal S1P_1_ activation in live mice. Three mouse genotypes were analyzed by BLI: heterozygous for the S1P_1_–CLuc allele (S1P_1_–CLuc); homozygous for the NLuc–β-arrestin2 allele (NLuc–β-arrestin2); and heterozygous for the S1P_1_–CLuc allele and homozygous for the NLuc–β-arrestin2 allele (S1P_1_LS). **g** A representative image comparing the basal bioluminescence activity in the three mouse lines. Mice were imaged in the supine position. Red open rectangles representing regions of interest (ROI) were positioned around cephalic, thoracic, and epigastric regions. **h** The bioluminescence activity was quantified by determining the total flux (photons/sec; p/sec) in each ROI. Data represent the mean ± SEM. *n* = 5 for S1P_1_–CLuc, *n* = 8 for S1P_1_LS, *n* = 5 for NLuc–β-arrestin2. **i** Basal S1P_1_ activation in internal organs. S1P_1_ luciferase signaling (S1P_1_LS) mice (*n* = 3). A representative image is shown (supine view). Arrow lymph node, Li liver, Sp spleen, Lu lung. *P* values were determined by one-way ANOVA followed by Tukey’s multiple comparisons test; **P ≤ 0.01, ***P ≤ 0.001

Our strategy for imaging the activation of a GPCR in real time in living mice was to adapt a firefly split luciferase complementation system^21,23,24^ for the detection of the interaction between ligand-activated S1P_1_ and β-arrestin2 (Fig. 1a). Both S1P_1_ and β-arrestin2 were genetically modified as individual fusion proteins to carry inactive, but complementary, fragments of luciferase. The S1P_1_ C terminus was linked to the C-terminal fragment of firefly luciferase (CLuc: amino acids #394–550), and the β-arrestin2 N terminus was linked to the N-terminal fragment of firefly luciferase (NLuc: amino acids #1–416). In this scheme, S1P_1_ activation will promote its interaction with β-arrestin2, facilitating the association of the inactive luciferase fragments and producing an active enzyme complex that, in the presence of substrates ATP and D-luciferin, generates light that can be detected by bioluminescence imaging (BLI).

The efficacy and specificity of the split luciferase complementation system for detection of S1P_1_ activation was tested by co-transfection of expression vectors containing the S1P_1_–CLuc and NLuc–β-arrestin2 fusion genes into U2OS cells. As a negative interaction control, NLuc was fused to herpes simplex virus thymidine kinase (HSV-tk) in place of β-arrestin2, and co-transfected with S1P_1_–CLuc. In transfected cells treated with vehicle, minimal luciferase activity was detected (Supplementary Fig. 1a, b). However, addition of S1P or RP-001, a synthetic S1P_1_-selective agonist^25^, caused a significant increase in luciferase activity in the S1P_1_–CLuc/NLuc–β-arrestin2-transfected cells compared with the S1P_1_–CLuc/NLuc–HSV-tk-transfected cells. These results demonstrate that the split luciferase complementation system, utilizing S1P_1_ and β-arrestin fusions, can specifically detect S1P_1_ activation.

Gene targeting in embryonic stem cells was employed to produce two individual mouse lines, each carrying one of the fusion constructs (Supplementary Fig. 2a, b). One mouse line was generated with the S1P_1_–CLuc fusion knocked in to the S1P_1_ coding region. In this configuration, the native S1P_1_ promoter elements were maintained to allow for expression of the S1P_1_ fusion gene in the endogenous context. The other mouse line was established with the NLuc–β-arrestin2 fusion knocked in to the safe harbor *Rosa26* locus under control of the *Rosa26* promoter elements to provide a ubiquitous expression pattern, ensuring that NLuc–β-arrestin2 would be co-expressed along with S1P_1_–CLuc. The S1P_1_–CLuc knock-in mice were crossed with the NLuc–β-arrestin2 knock-in mice to derive mice carrying both alleles (Fig. 1a), which are termed S1P_1_ luciferase signaling mice.

To determine whether the synthetic S1P_1_ signaling pathway genetically encoded within the S1P_1_ luciferase signaling mice was responsive to ligands with specificity similar to native S1P_1_, primary mouse embryonic fibroblasts (MEFs) derived from S1P_1_ luciferase signaling mice were utilized. Addition of S1P but not vehicle rapidly induced bioluminescence in the MEFs (Fig. 1b). Bioluminescence activity peaked within 3 min after addition of S1P to the MEFs and was rapidly lost thereafter (Fig. 1b). This loss of activity indicates that luciferase fragment complementation is transient and may reflect the rapid kinetics of S1P_1_ desensitization after activation and the subsequent dissociation of the receptor–β-arrestin2 complex^26^. S1P and RP-001, a potent synthetic S1P_1_-selective agonist^25^, induced luciferase activity in the S1P_1_ luciferase signaling MEFs, but not in MEFs carrying only the S1P_1_–CLuc or the NLuc–β-arrestin2 alleles (Fig. 1b, c), showing that both components of the genetically encoded split luciferase complementation system were necessary to report the ligand-activated S1P_1_–CLuc interaction with NLuc–β-arrestin2. Two structural analogs of S1P, lysophosphatidic acid (LPA) and sphingosine, that are not ligands for S1P_1_ did not induce bioluminescence when added to S1P_1_ luciferase signaling MEFs at a concentration of 10^−6^ M (Fig. 1d, e).

The EC_50_ values for the activation of bioluminescence by S1P and RP-001 in S1P_1_ luciferase signaling MEFs were determined to be 19.4 ± 0.8 and 0.46 ± 0.02 nM, respectively (Fig. 1f). The natural ligand dihydro-S1P (dhS1P)^27^, exhibited an EC_50_ for the activation of bioluminescence of 32.4 ± 0.5 nM.

To determine if the genetically encoded split luciferase complementation system could detect basal S1P_1_ activation in vivo, S1P_1_ luciferase signaling mice, as well as mice carrying only the S1P_1_–CLuc or NLuc–β-arrestin2 alleles, were subjected to BLI while under anesthesia. Significantly higher levels of bioluminescence activity were detected in the S1P_1_ luciferase signaling mice compared with the mice carrying only the S1P_1_–CLuc or NLuc–β-arrestin2 alleles (Fig. 1g). We quantified bioluminescence activity in the cephalic, thoracic, and epigastric regions, as the activity varied between different body regions. The highest activity was observed in the epigastric region, followed by the thoracic and cephalic regions (Fig. 1h). To identify the anatomical sites of basal S1P_1_ activation in S1P_1_ luciferase signaling mice, S1P_1_ luciferase signaling mice were subjected to BLI after surgically exposing the thoracic region and abdominal cavity. The highest bioluminescence signals were detected in lungs, in lymph nodes, and in the splenic region. Notably, no signal was detected in the liver (Fig. 1i).

### Treatment of S1P_1_ signaling mice with antagonist

**Fig. 2:**
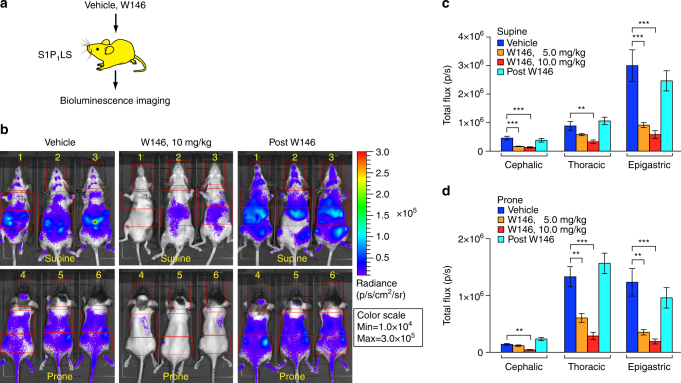
S1P_1_ activation after treatment with an S1P_1_ antagonist in live mice. **a** S1P_1_ luciferase signaling (S1P_1_LS) mice were serially injected with vehicle, followed by the S1P_1_ antagonist W146 (5 or 10 mg/kg, ip injection). **b** Representative repeat bioluminescence images of the same mice (top, supine view of mouse # 1, 2, 3; bottom, prone view of mouse # 4, 5, 6) comparing the effects of vehicle with those of W146 (10 mg/kg), 0.5 h after injection. BLI was also performed 1 day after the W146 injection (post W146). Red open rectangles representing ROI were positioned around cephalic, thoracic, and epigastric regions. **c**, **d** The bioluminescence activity was quantified by determining the total flux (photons/sec; p/sec) in each ROI. Data acquired from supine (**c**) and prone (**d**) views are shown. Data represent the mean ± SEM. *n* = 6 for each group. *P* values were determined by one-way ANOVA followed by Bonferroni’s multiple comparisons test; **P ≤ 0.01, ***P ≤ 0.001

To determine if the bioluminescence activity detected in S1P_1_ luciferase signaling mice was the result of endogenous S1P_1_ activation, the mice were injected with W146^28^, a potent S1P_1_-selective antagonist (Fig. 2a). W146 treatment administered 30 min prior to BLI significantly lowered the bioluminescence in the S1P_1_ luciferase signaling mice by 70–80% in a dose-dependent manner in the cephalic, epigastric, and thoracic regions compared with the same mice treated with vehicle (Fig. 2b–d). The basal bioluminescence activity was completely restored in these S1P_1_ luciferase signaling mice 1 day post-W146 injection (Fig. 2b–d), which is consistent with the rapid clearance reported for this compound^29^. The inhibition of bioluminescence activity by an S1P_1_ antagonist indicates that the basal activity detected in the live mice is primarily the result of endogenous S1P_1_ activation.

### Treatment of S1P_1_ signaling mice with agonists

**Fig. 3:**
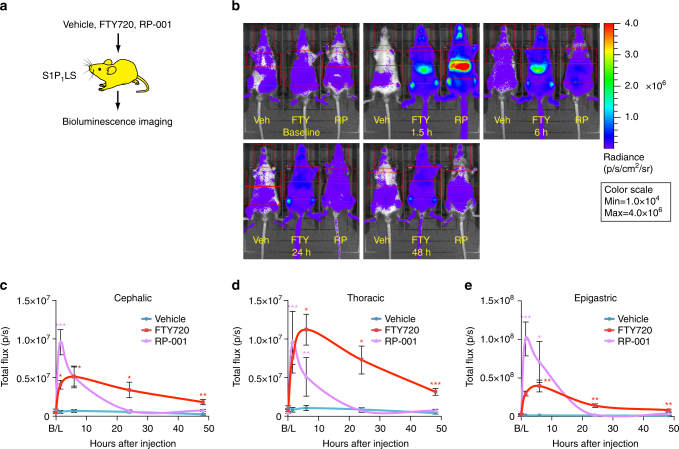
S1P_1_ activation after treatment with S1P_1_ agonists in live mice. **a** S1P_1_ luciferase signaling (S1P_1_LS) mice were intraperitoneally injected with vehicle or the S1P_1_ agonists FTY720 or RP-001, then subjected to BLI. **b** Mice were subjected to imaging prior to injection (baseline, B/L) and 1.5, 6, 24, and 48 h after injection. Representative bioluminescence images of the same three mice comparing the effects of vehicle (Veh), FTY720 (FTY), or RP-001 (RP) at the specified time points. Mice were imaged in the supine position. Red open rectangles representing ROI were positioned around cephalic, thoracic, and epigastric regions. **c**–**e** The bioluminescence was quantified by determining the total flux (photons/sec; p/sec) in the cephalic (**c**), thoracic (**d**), and epigastric (**e**) ROI. Data represent the mean ± SEM. *n* = 5 for vehicle, *n* = 8 for FTY720, *n* = 6 for RP-001. *P* values between vehicle and agonist-injected groups were determined by one-way ANOVA followed by Bonferroni’s multiple comparisons test; *P ≤ 0.05, **P ≤ 0.01, ***P ≤ 0.001

FTY720 (fingolimod/Gilenya^TM^), a sphingosine analog, is phosphorylated by sphingosine kinases in vivo, and converted to a form that, at nanomolar concentrations, activates S1P_1_, as well as S1P_3_, S1P_4_, and S1P_5_^10^. Its half-life in blood after oral administration in rats is ~24 h^30^. To determine the time course of S1P_1_ activation by FTY720 in the S1P_1_ luciferase signaling mice, the mice were imaged prior to the administration of compounds and then imaged serially at 1.5, 6, 24, and 48 h after intraperitoneal injection of each compound (Fig. 3). FTY720 concurrently induced peak levels of bioluminescence activity in the cephalic, thoracic, and epigastric regions at 6 h after injection. Significant bioluminescence activity continued to be detected at 24 and 48 h after injection of FTY720, commensurate with the relatively long half-life of the drug. BLI performed on FTY720-treated S1P_1_ luciferase signaling mice (5 h after FTY720 treatment) with their internal organs surgically exposed showed highly elevated bioluminescence signals over lung, lymph nodes, spleen, and liver (Supplementary Fig. 3a). Compared with FTY720, RP-001 is a very short-acting S1P_1_ agonist that decreases to undetectable levels in blood by 8 h after administration in mice^25^ but is substantially more potent, being effective at picomolar concentrations. RP-001 induced a peak of bioluminescence activity 1.5 h after administration to S1P_1_ luciferase signaling mice in the cephalic, thoracic, and epigastric regions. The bioluminescence signal was most intense in the epigastric region. By 24 and 48 h after dosing, the bioluminescence activity in RP-001-treated S1P_1_ luciferase signaling mice declined to baseline levels (Fig. 3b–e).

### LPS-induced systemic inflammation in S1P_1_ signaling mice

**Fig. 4:**
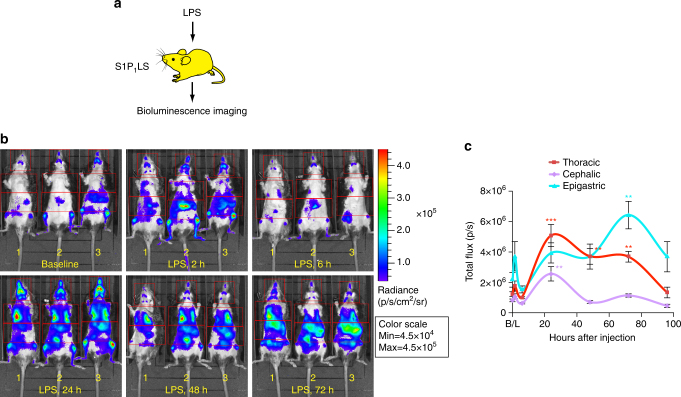
S1P_1_ activation during LPS-induced systemic inflammation in live mice. **a** S1P_1_ luciferase signaling (S1P_1_LS) mice were injected intraperitoneally with LPS to induce systemic inflammation, then subjected to BLI. **b** Representative bioluminescence images of the same three mice prior to LPS injection (baseline, B/L) and 2, 6, 24, 48, and 72 h after LPS injection. Mice were imaged in the supine position. Red open rectangles representing ROI were positioned around cephalic, thoracic, and epigastric regions. **c** The bioluminescence activity was quantified by determining the total flux (photons/sec; p/sec) in each ROI. Data represent the mean ± SEM. *n* = 5. *P* values were determined between baseline and each time point by one-way ANOVA followed by Bonferroni’s multiple comparisons test; **P ≤ 0.01, ***P ≤ 0.001

S1P_1_ signaling is activated during systemic inflammation induced by bacterial lipopolysaccharide (LPS); however, the timing and anatomical distribution of receptor activation is not well established^22^. In order to define these parameters, S1P_1_ luciferase signaling mice were intraperitoneally injected with a sublethal dose of LPS and then subjected to serial BLI at 2, 6, 24, 48, 72, and 96 h (Fig. 4). At 2 h after injection, a weak increase in bioluminescence was observed primarily in the epigastric region, which returned to baseline levels at 6 h. At 24 h after administration, the bioluminescent signal was significantly increased in the cephalic and thoracic regions (Fig. 4b, c and Supplementary Fig. 5). At 48 h after administration, bioluminescent signal was elevated in the thoracic region. At 72 h after administration, the strongest signal was observed in the epigastric region. At 24 h after LPS administration, BLI performed in S1P_1_ luciferase signaling mice with surgically exposed internal organs demonstrated bioluminescent signals induced over lungs, lymph nodes, spleen, and liver (Supplementary Fig. 3b). These results show that LPS induces heightened S1P_1_ activation in a sustained manner systemically. In addition, the timing of S1P_1_ activation was distinctive for specific anatomical locations.

### LPS-induced S1P_1_ activation by hematopoietically derived S1P

**Fig. 5:**
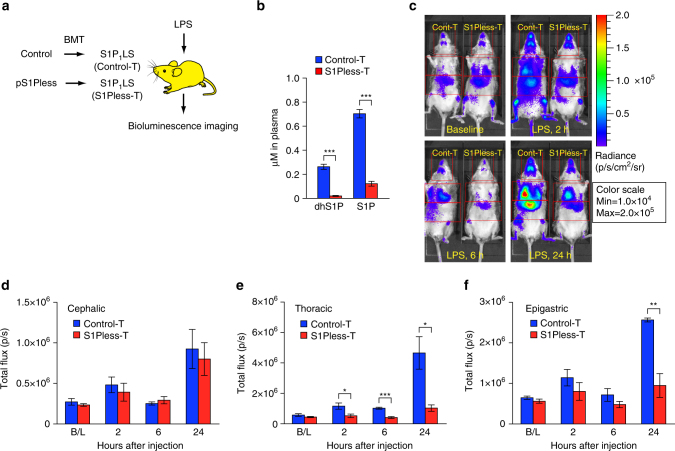
S1P_1_ activation during LPS-induced systemic inflammation in live mice transplanted with plasmaS1Pless bone marrow. **a** Bone marrow cells from control or plasma(p)S1Pless mice were transplanted (BMT) into irradiated S1P_1_ luciferase signaling (S1P_1_LS) mice to produce control transplanted (Control-T) or pS1Pless transplanted (S1Pless-T) mice, respectively. LPS was injected intraperitoneally into the stably transplanted mice 20 weeks later. Mice were subjected to BLI prior to the LPS injection (baseline, B/L) and 2, 6, and 24 h after LPS injection. **b** Plasma S1P and dhS1P levels in S1P_1_ luciferase signaling mice, transplanted with control (Control-T) or pS1Pless (S1Pless-T) bone marrow. Data represent the mean ± SEM. *n* = 6 for Control-T mice, *n* = 8 for S1Pless-T mice. **c** Representative bioluminescence images of the same S1P_1_ luciferase signaling mice, transplanted with control (Cont-T) or pS1Pless (S1Pless-T) bone marrow, at the specified time points. Mice were imaged in the supine position. Red open rectangles representing ROI were positioned around cephalic, thoracic, and epigastric regions. **d**–**f** The bioluminescence activity was quantified by determining the total flux (photons/sec; p/sec) in the cephalic (**d**), thoracic (**e**), and epigastric (**f**) ROI. Data represent the mean ± SEM. *n* = 5 for control BM-transplanted S1P_1_ luciferase signaling (Control-T) mice and *n* = 7 for pS1Pless BM-transplanted S1P_1_ luciferase signaling (S1Pless-T) mice at the baseline, 2 h, and 6 h time points. *n* = 4 for control BM-transplanted S1P_1_ luciferase signaling (Control-T) mice and *n* = 6 for pS1Pless BM-transplanted S1P_1_ luciferase signaling (S1Pless-T) mice at 24 h. *P* values were determined two-tailed Student’ *t*-test; *P ≤ 0.05, **P ≤ 0.01, ***P ≤ 0.001

A major fraction of circulating S1P is produced by hematopoietically derived cells^31^. To determine if hematopoietic cell-derived S1P is critical for systemic S1P_1_ activation during LPS-induced inflammation, irradiated S1P_1_ luciferase signaling mice were transplanted with sphingosine kinase-deficient bone marrow from plasmaS1Pless mice, which lack the ability to produce S1P in the hematopoietic system^31^, and then subjected to BLI (Fig. 5a). Plasma S1P and dhS1P levels were significantly decreased in the plasmaS1Pless bone marrow-transplanted mice to ~20 and 10%, respectively, of the levels observed in control bone marrow-transplanted mice (Fig. 5b). The residual S1P and dhS1P in the plasmaS1Pless bone marrow-transplanted mice are likely produced by the sphingosine kinase-replete endothelial cells of the recipient mice^32^. After LPS treatment, mice transplanted with control bone marrow exhibited significantly increased bioluminescence activity compared with the plasmaS1Pless bone marrow-transplanted mice in the thoracic region at 2, 6, and 24 h and in the epigastric region at 24 h (Fig. 5c–f). The residual bioluminescence activity in plasmaS1Pless bone marrow-transplanted mice at 24 h after LPS treatment was significantly inhibited by the S1P_1_ antagonist W146 (Supplementary Fig. 4). These results suggest that the low levels of dhS1P and S1P remaining in these mice may provide some signaling activity. A high degree of mortality occurred 24 h after LPS treatment in S1P_1_ luciferase signaling mice transplanted with plasmaS1Pless bone marrow, precluding further measurements. These results indicate that hematopoietically derived S1P is responsible for a significant portion of the S1P_1_ activation that occurs in vivo during LPS-induced inflammation.

### S1P_1_ activation in the brain by inflammation

**Fig. 6:**
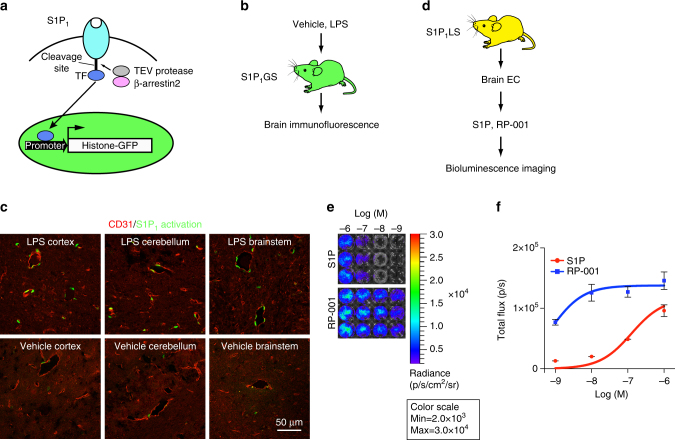
S1P_1_ activation by LPS in the brain. **a** Schematic of the design to monitor S1P_1_ activation in S1P_1_ GFP signaling mice. S1P_1_ is fused to a transcription factor (TF) via a protease recognition sequence. Ligand binding to the receptor stimulates the recruitment of a β-arrestin2–TEV protease fusion protein, triggering the release of the transcription factor from the C terminus of modified S1P_1_. The transcription factor stimulates expression of a histone-GFP reporter gene, fluorescently labeling cell nuclei. **b** LPS or vehicle was injected intraperitoneally into S1P_1_ GFP signaling (S1P_1_GS) mice, and the brains were harvested 7 days after injection and examined by immunofluorescence. The tissues of three mice injected with LPS and three mice injected with vehicle were examined. **c** Representative histological sections were immunostained with antibodies to CD31, and the images of cerebral cortex, cerebellum, and brainstem were captured using an inverted laser scanning confocal microscope. Scale bar, 50 μm. **d** Brain endothelial cells (EC) were isolated from S1P_1_ luciferase signaling (S1P_1_LS) mice, grown in triplicate in 24-well plates, treated with S1P or RP-001 and D-luciferin, and immediately subjected to BLI. **e** Bioluminescence images of the cells exposed to the indicated concentrations of S1P or RP-001 represent a 15-min acquisition. The experiment was repeated twice. **f** The bioluminescence activity was quantified by determining the total flux (photons/sec; p/sec). Data represent the mean ± SEM, *n* = 6

During LPS-induced inflammation, bioluminescence activity was significantly increased in the cephalic region at 24 h (Fig. 4b, c and Supplementary Fig. 5), raising the possibility that S1P_1_ may be activated in the central nervous system (CNS). To determine if S1P_1_ signaling was activated within the CNS and, if so, in which cell types, we utilized the previously described S1P_1_ GFP signaling mice^22^. In these mice, S1P_1_ activation is also based on interaction of the GPCR with β-arrestin2; however, in this reporter system the interaction leads to the proteolytic release of a transcription factor tethered to S1P_1_ that enters the nucleus and activates a histone-GFP reporter gene. The cells in which S1P_1_ activation occurs are then stably marked with GFP-labeled nuclei (Fig. 6a). LPS was administered to S1P_1_ GFP signaling mice and, 7 days later, brain tissue was collected and sectioned, then the cortex, cerebellum, and brainstem were examined for GFP-labeled cells (Fig. 6b, c). Compared with vehicle-treated mice, LPS-treated mice exhibited increased numbers of GFP-positive cells in each of these regions, indicating that S1P_1_ activation had taken place in the CNS. The GFP-positive cells appeared to be associated with the vasculature and were CD31 positive, establishing their identity as endothelial cells (Fig. 6c). Next, brain endothelial cells were isolated from S1P_1_ luciferase signaling mice, treated with S1P or RP-001, and examined by BLI. Bioluminescent activity was induced in the brain endothelial cells in a dose-dependent manner, with RP-001 exhibiting a higher potency than S1P (Fig. 6d–f). Collectively, the results indicate that S1P_1_ is activated in endothelial cells of the neurovascular unit of the brain during systemic inflammation induced by LPS and illustrate that the two different S1P_1_ signaling mouse models provide complementary information for defining S1P_1_ activation in vivo.

## Discussion

The luciferase signaling model for GPCR activation described here affords a way to study GPCRs that was not previously possible, yielding spatial and temporal information on the status of endogenous receptor activation in real time in a living animal. The nearly universal interaction between GPCRs with β-arrestin^13^ was exploited to induce assembly of inactive fragments of luciferase, producing an active enzyme complex and enabling BLI upon activation of a GPCR, S1P_1_, in living mice. The in vivo S1P_1_ signaling detection system was shown to be activated by both synthetic and natural ligands of S1P_1_. BLI of the luciferase signaling mice revealed the anatomical locations of homeostatic S1P_1_ signaling, as well as the dynamics of S1P_1_ signaling under inflammatory conditions. Although the resolution of the luciferase signaling model was limited to the identification of signaling at an organ or tissue level, the associated use of the previously described GFP signaling model for S1P_1_ activation^22^, which relies on transcription factor reporter gene activation, provided a means to ultimately define S1P_1_ signaling sites at a cellular level. While visualizing the source of the bioluminescent signal with single cell resolution is not possible at present, detection of signaling in real-time in vivo at a single cell level would be an important goal to achieve.

We have demonstrated that the S1P_1_ luciferase signaling mice can be used for the characterization of the in vivo signaling activity of the receptor-active pharmacological compounds FTY720 and RP-001 (Fig. 3). FTY720^10^, an approved drug (fingolimod/Gilenya^TM^) used for the treatment of multiple sclerosis, targets S1P receptors 1, 3, 4, and 5. It has a relatively long half-life in blood^30^ and has been demonstrated to have diverse physiologic effects in the immune, cardiovascular, and nervous systems^7^. RP-001 is an S1P_1_-selective agonist that is more potent than FTY720, but with a much shorter half-life in blood. Similar to FTY720, RP-001 has been shown to trigger lymphopenia^25^. Administration of these compounds to the S1P_1_ luciferase signaling mice enabled determination of the timing of signaling and anatomical location of signaling events, which indicates that this model will be generally useful for characterization of S1P_1_ modulators. The signaling induced by RP-001 was relatively intense but decayed rapidly, commensurate with its high potency and short half-life (Fig. 3b–e). The very intense epigastic signal induced by RP-001 may reflect its rapid clearance in the liver. In contrast, the signaling induced by FTY720 in vivo persisted over a long duration, lasting up to 48 h (Fig. 3b–e). While consistent with the relatively long half-life of the compound, prolonged signaling, and the apparent absence of rapid desensitization would be compatible with the existence of a receptor reserve. Under normal conditions, a receptor reserve may underpin the long-term tonic S1P_1_ signaling in the vascular endothelium. Compartmentalization of S1P_1_ receptors may be a mechanism underlying such a receptor reserve^33,34^. However, a different mechanism must operate on other cell types, such as lymphocytes, that rapidly downmodulate S1P_1_ upon exposure to agonist^25^.

The S1P_1_ luciferase signaling mice provide the unique capability to detect the signaling activity of the endogenously produced natural ligand under basal and pathophysiological conditions in real time. Under basal conditions, lung and lymphoid tissues showed the highest levels of signaling (Fig. 1i). This is compatible with ample evidence for a functional S1P_1_ signaling requirement in these tissues. In lung, blocking S1P_1_ signaling—either by removal of S1P from the blood^35^ or by S1P_1_ antagonism^28,29^—induced vascular leakage, indicating that S1P_1_ signaling is critical to maintain vascular integrity in lung. In lymphoid tissues, S1P_1_ signaling has been shown to be necessary for lymphocyte egress^36^ and for the positioning of cells, such as splenic marginal zone B cells^37^. S1P_1_ signaling is also required in endothelial cells in lymph nodes, notably on high endothelial venules, which serve as the portals for lymphocyte entry to maintain their vascular integrity^38^.

Under conditions of systemic inflammation induced by LPS exposure, the S1P_1_ luciferase signaling mice reported region-specific dynamics in S1P_1_ activation (Fig. 4). Liver showed little or no signaling activity under basal conditions (Fig. 1i), but exhibited dramatically increased S1P_1_ signaling during systemic inflammation (Supplementary Fig. 3b). Hepatocytes have been shown to express abundant S1P_1_ that is accessible for signaling in response to FTY720 administration^22^. Based on the findings reported here, they are apparently not exposed to sufficient S1P under basal conditions to stimulate signaling. However, during conditions that induce vascular leakage, such as inflammation, S1P derived from blood sources can stimulate S1P_1_ signaling in hepatocytes and endothelial cells of liver, and endothelial cells in lung, as has been demonstrated in an S1P_1_ GFP signaling mouse model^22^. We have extended these finding here by showing increased S1P_1_ signaling in brain endothelial cells during systemic inflammation (Fig. 6).

The strategy used to produce the S1P_1_ luciferase signaling mice provides a template for the development of a library of other GPCR-signaling models. Creation of models for other GPCRs that similarly interact with β-arrestin2 should only require the derivation of a mouse with a receptor knock-in of the C-terminal Luc fragment (amino acids #394–550). Subsequent cross-breeding with the NLuc–β-arrestin2 mouse described here, which carries the complementary luciferase N-terminal fragment (amino acids #1–416), would yield unique GPCR-signaling models. These models can be used to gain new understandings of GPCR signaling in normal and disease-specific biological contexts, and enable in vivo analysis of GPCR-signaling perturbation by receptor-active compounds, thereby facilitating drug development for this important target class.

## Methods

### Reagents

S1P (d18:1), sphingosine (d18:1), and LPA (18:1) were purchased from Avanti Polar Lipids; dhS1P (d18:0), LPS (*Escherichia coli* 055:B5; L2880), and polyinosinic–polycytidylic ribonucleic acid (pIpC) (P0913) from Sigma-Aldrich; FTY720 and W146 from Cayman; and RP-001 from Tocris.

### Generation of S1P_1_ luciferase signaling mice

A split firefly luciferase enzyme complementation system^23,24^ (Fig. 1a) was designed for detection of the S1P_1_ and β-arrestin2 interaction after receptor activation. The fragments were fused to the mouse *S1pr1* (Accession: NM_007901.5) or *Arrb2* (Accession: NM_145429.5) coding regions via a linker (GGGGSGGGGS); the *S1pr1* coding region was linked to the C-terminal luciferase fragment (amino acids #394–550) to produce the S1P_1_–CLuc fusion and the *Arrb2* coding region was linked to the N-terminal luciferase fragment (amino acids #1–416) to produce the NLuc–β-arrestin2 fusion. Targeting vectors for mouse embryonic stem cells were constructed to insert the S1P_1_–CLuc or NLuc–β-arrestin2 fusions into the *S1pr1* or *Rosa26* locus, respectively (Supplementary Fig. 2a, b). In the *S1pr1*-targeting vector, the neomycin resistance gene (NeoR), flanked by loxP sites, was inserted downstream of the fusion gene. In the *Rosa26*-targeting vector^39^, NeoR and a stop cassette (NeoR Stop), flanked by loxP sites, was placed upstream of the fusion gene. Gene targeting in embryonic stem cells and generation of chimeric and heterozygous mice were conducted as described previously^22^. The genotypes were determined by PCR analysis of genomic DNA isolated from mouse tails. For genotyping of the S1P_1_–CLuc fusion knock-in mice carrying NeoR, three primers were used: 5′-AGAGGAATGTGGGCTGTTGATCCT-3′ (Primer 1); 5′-GGTGAACATCCACCCACTATTCCA-3′ (Primer 2); and 5′-CCAAATTAAGGGCCAGCTCATTCC-3′ (Primer 3). Primers 1 and 2 detected the wild-type (WT) allele and amplified a 290-bp fragment. Primers 2 and 3 detected the knock-in allele carrying NeoR and amplified a 400-bp fragment. Forty cycles of 94 °C (1 min), 60 °C (1 min), and 72 °C (1.5 min) were used for PCR. For genotyping of the NLuc–β-arrestin2 knock-in mice bearing NeoR stop, three primers were used: 5′-GCACTTGCTCTCCCAAAGTC-3′ (Primer 4); 5′-GGCGGATCACAAGCAATAAT-3′ (Primer 5); and 5′-TACCGGTGGATGTGGAATGT-3′ (Primer 6). Primers 4 and 5 detected the WT allele and amplified a 450-bp fragment. Primers 4 and 6 detected the knock-in allele bearing NeoR and the stop codon and amplified a 580-bp fragment. Forty cycles of 94 °C (1 min), 63 °C (1 min), and 72 °C (1.5 min) were used for PCR.

An EIIa Cre recombinase transgenic line^40^ (stock #003724, The Jackson Laboratory) was crossed with the knock-in mice to remove the loxP-flanked genes in the germline. For genotyping of the S1P_1_–CLuc knock-in mice with NeoR excised (–NeoR), three primers were used. Primers 1 and 7 (5′-AGATGGCGGTAACTCTGAGG-3′) detected the WT allele and amplified a 400-bp fragment. Primers 7 and 8 (5′-TAGTTGCCAGCCATCTGTTGTTTG-3′) detected the S1P_1_–CLuc (–NeoR) knock-in allele and amplified a 510-bp fragment. Forty cycles of 95 °C (1 min), 63 °C (1 min), and 72 °C (1.5 min) were used for PCR. For genotyping of the NLuc–β-arrestin2 (–NeoR Stop) knock-in mice, three primers were used. Primers 4 and 5 detected the WT allele as described above, and primers 4 and 9 (5′-CCGTCTTCGAGTGGGTAGAA-3′) detected the NLuc–β-arrestin2 (–NeoR Stop) knock-in allele and amplified a 560-bp fragment. Forty cycles of 95 °C (1 min), 63 °C (1 min), and 72 °C (1.5 min) were used for PCR.

The scheme to derive S1P_1_ luciferase signaling mice is shown in Fig. 1a. Mice heterozygous for S1P_1_–CLuc (–NeoR) knock-in mice were crossed with mice heterozygous for NLuc–β-arrestin2 (–NeoR) knock-in mice to obtain double heterozygotes, which were back crossed to mice heterozygous for NLuc–β-arrestin2 (–NeoR) to obtain mice heterozygous for S1P_1_–CLuc (–NeoR) and homozygous for NLuc–β-arrestin2 (–NeoR). S1P_1_ luciferase signaling mice that were heterozygous for the S1P_1_–CLuc (–NeoR) allele and homozygous for the NLuc–β-arrestin2 (–NeoR Stop) allele showed a higher bioluminescence signal after FTY720 treatment than mice heterozygous for both of these alleles (Supplementary Fig. 6). For this reason, mice heterozygous for the S1P_1_–CLuc (–NeoR) allele and homozygous for the NLuc–β-arrestin2 (–NeoR stop) allele were used in S1P1 luciferase signaling mouse studies. Animals were housed in a specific pathogen-free facility and provided food and water ad libitum. Age-matched offspring of both sexes (2–5 months old) were used for experiments. The mice were at the initiation of experiments. The sample size was selected based on expected effect size. No randomization method was employed. Investigators were blinded to the genotype of animals during data analysis. No experimental animals were excluded from the analysis.

### BLI of S1P_1_ activation in cells and mice

S1P_1_–CLuc, NLuc–β-arrestin2, and NLuc–HSV-tk (accession: Q9QNF7.1) fusions were prepared using a linker (GGGGSGGGGS) sequence as described above and cloned into pcDNA3.1 (Thermo Fisher Scientific). U2OS cells (American Type Culture Collection) were plated in 24-well plates (1.5 × 10^5^ cells/well) and transfected by pcDNA3.1-S1P_1_–CLuc (10 ng) together with pcDNA3.1-NLuc–β-arrestin2 (500 ng) or pcDNA3.1-NLuc–HSV-tk (500 ng) using Lipofectamine 3000 (Thermo Fisher Scientific). The media was replaced 24 h after transfection with DMEM containing 10% charcoal-stripped FBS and the cells incubated for 16 h. The media was then changed to DMEM with 0.1% FBS for 4 h, and then treated simultaneously with 150 μg/ml D-luciferin (K + Salt Bioluminescent Substrate, PerkinElmer) and S1P, RP-001, or vehicle. Bioluminescence activity was immediately detected with an in vivo imaging system (IVIS Lumina II, PerkinElmer).

S1P_1_ luciferase signaling MEFs were isolated from E12.5 embryos with three different genotypes: heterozygous for the S1P_1_–CLuc allele; homozygous for the NLuc–β-arrestin2 allele; and heterozygous for the S1P_1_–CLuc allele and homozygous for the NLuc–β-arrestin2 allele (S1P_1_ luciferase signaling mice). The MEFs were plated in 24-well plates (2 × 10^5^ cells/well) and incubated for 16 h in DMEM containing 10% charcoal-stripped FBS for 16 h followed by DMEM with 0.1% FBS for 4 h. After treatment with agonists, bioluminescence activity was measured immediately as described above.

S1P_1_ luciferase signaling mouse brain endothelial cells were prepared as previously described^41^. The endothelial cells in 24-well plates (10^5^/well) were incubated in medium (DMEM high glucose [12–709 F, LONZA], 5 μg/ml endothelial cell growth factor [BT-203, Biomed Tech], 100 μg/ml heparin, 100 U/ml penicillin, and 100 U/ml streptomycin) containing 10% charcoal-stripped FBS for 16 h followed by medium containing 0.1% BSA for 4 h. After treatment with agonists, bioluminescence activity was immediately detected as described above.

For BLI in live mice, the hair was removed with a clipper and hair removal cream. XenoLight Rediject D-luciferin (PerkinElmer) (150 mg/kg BW) was injected intraperitoneally, and the mice were subjected to BLI using an in vivo imaging system (IVIS Lumina II). During the imaging, the mice were anesthetized with isoflurane (Baxter) using an XGI-8 gas anesthesia system (PerkinElmer). Eye lubricant (Puralube Vet Ointment, Dechra) was applied during the anesthesia. Imaging parameters were: binning = medium (8), field of view = 12.5 × 12.5 cm, f/stop = 1. Each image exposure lasted 5 min, and a total of four sequential images were captured. The second 5-min image was used in all experiments. Images were analyzed using Living Image Software (PerkinElmer). Regions of interest (ROI) were drawn around each body region and the total flux (photons/s) was determined.

For BLI of internal organs, mice were anesthetized by intraperitoneal injection of ketamine (180 mg/kg BW)—xylazine (12 mg/kg BW), and after 15 min received an intraperitoneal XenoLight Rediject D-luciferin (PerkinElmer) injection (500 mg/kg BW). The ventral skin was incised along the midline to expose the internal organs. Bioluminescence activity was immediately detected with an in vivo imaging system (IVIS Lumina II) with four acquisitions lasting 5 min each.

In some mouse studies, prior to imaging, mice were intraperitoneally injected with FTY720 (1 mg/kg BW; vehicle, 43% ethanol-7% DMSO in PBS), RP-001 (1 mg/kg BW; vehicle, 43% ethanol-7% DMSO in PBS), W146 (5 and 10 mg/kg BW; vehicle, 10 mM sodium carbonate-2% (2-hydroxypropyl)-β-cyclodextrin in water), or LPS (16 mg/kg BW; vehicle, PBS).

### Analysis of S1P_1_ activation in brain

S1P_1_ GFP signaling mice have been previously described^22^. LPS (16 mg/kg BW; vehicle, PBS) was injected intraperitoneally into S1P_1_ GFP signaling mice. Seven days after the injection, the mice were perfused with normal saline followed by chilled 4% paraformaldehyde in 0.1 M phosphate buffer (pH 7.4) and the brains were harvested. The tissues were post fixed in 4% paraformaldehyde for 4 h and incubated in 30% sucrose in 0.1 M phosphate buffer (pH 7.4) at 4 °C for 2 days, then embedded in OCT (Sakura) and sectioned at 7-μm thickness. For immunostaining, nonconjugated primary antibody for mouse CD31 (clone SZ31, Dianova) and fluorescently labeled secondary antibody (donkey anti-rat IgG Cyanine Cy3, Jackson ImmunoResearch Laboratories) were used. The images were captured with an inverted laser scanning confocal microscope (LSM 780, Carl Zeiss Microscopy) using Zeiss Zen 2012 software.

### Preparation of plasma S1P-deficient S1P_1_ luciferase signaling mice

*Sphk1* ^*flox*/*flox*^ *Sphk2* ^−/−^ Mx1-cre mice were obtained by breeding *Sphk1* ^*flox*/*flox*^ mice (stock #030038-UCD, MMRRC), *Sphk2* ^−/−^ mice^42^ and Mx1-cre mice^43^ (stock #003556, The Jackson Laboratory). Plasma S1P-deficient (plasmaS1Pless) mice were prepared by injection of pIpC into *Sphk1* ^*flox*/*flox*^ *Sphk2* ^−/−^ Mx1-cre pups as described^31^. Controls (*Sphk1* ^*flox*/*flox*^ *Sphk2* ^−/−^) were similarly bred and injected with pIpC. Bone marrow cells were isolated from the femurs and tibias of adult control and plasmaS1Pless mice 4 weeks after pIpC injection. A single cell suspension of control or plasmaS1Pless bone marrow cells (1 × 10^7^ cells/mouse) in 0.2 ml saline was injected into the tail vein of irradiated (9 Gy) S1P_1_ luciferase signaling mice. These mice, termed control and plasmaS1Pless S1P_1_ luciferase signaling mice, respectively, were used 20 weeks after transplantation.

### Sphingolipid analysis

S1P and dhS1P were measured by HPLC-tandem MS by the Lipidomics Core at the Medical University of South Carolina on a Thermo Finnigan TSQ 7000 triple quadrupole mass spectrometer (Thermo Fisher Scientific), operating in a multiple reaction monitoring-positive ionization mode as described^44^.

### Statistical analysis

GraphPad Prism v.6.0 software was used for statistical analyses. To determine the significance between three or more test groups, analysis of variance (ANOVA) was used followed by either Bonferroni’s for comparison to the control group or Tukey’s to compare all groups. Two-tailed Student’s *t*-test was used for the direct comparison of two groups. In all cases, *P* ≤ 0.05 was considered statistically significant.

### Study approval

All animal procedures were approved by the Animal Care and Use Committee of the National Institute of Diabetes and Digestive and Kidney Diseases and performed in accordance with the National Institutes of Health guidelines.

### Data availability

The authors declare that all data supporting the findings of this study are available within this paper or its Supplementary Information file or are available from the corresponding author on request.

## Electronic supplementary material

Supplementary Information
