# Supplementary material for: Bioluminescence imaging of G protein-coupled receptor activation in living mice
Source: Nat Commun. 2017 Oct 27;8:1163. doi: 10.1038/s41467-017-01340-7 (PMC5660082; doi:10.1038/s41467-017-01340-7)
Supplement: Supplementary file 1 — Supplementary Information [file 41467_2017_1340_MOESM1_ESM.pdf]

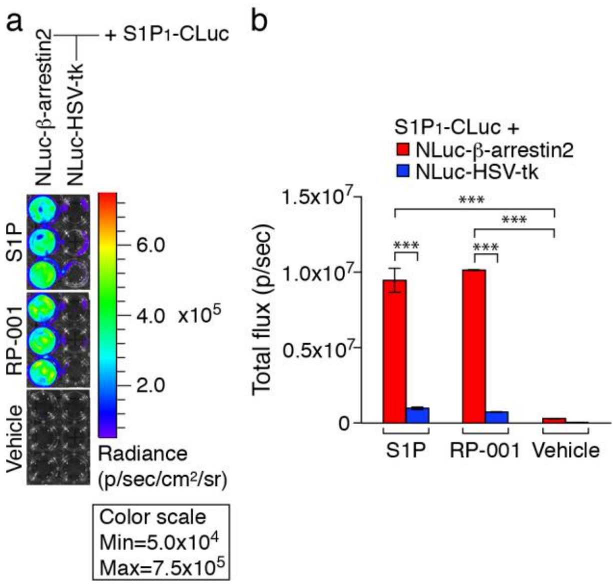

Supplementary Figure 1. Specificity of the agonist-induced $S1P_1$-CLuc and NLuc–β-arrestin2 interaction. a, U2OS cells were plated in 24-well plates, transfected by pcDNA3.1-$S1P_1$-CLuc (10 ng) with pcDNA3.1-NLuc–β-arrestin2 (500 ng) or pcDNA3.1-NLuc–HSV-tk (500 ng), treated with S1P, RP-001 (10^{-6} M), or vehicle and D-luciferin, then immediately subjected to BLI. Imaging was for a 3-min period. The experiment was repeated twice and representative images are shown. **b,** The bioluminescence activity was quantified by determining the total flux (photons/sec; p/sec). Data represent the mean ± SEM, n=3. *P*-values were determined by one-way ANOVA followed by Tukey's multiple comparisons test); ***, $P \leq 0.001$.

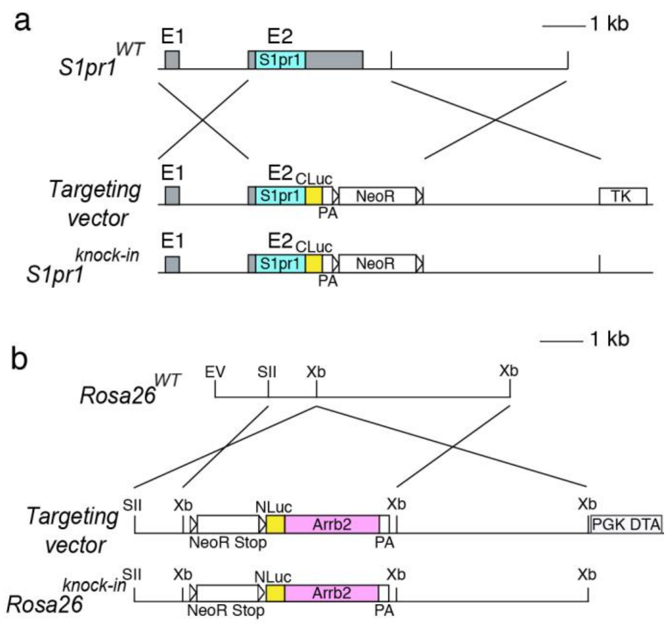

Supplementary Figure 2. Gene targeting to produce S1P$_1$-CLuc and NLuc–β-arrestin2 knock-in alleles in mouse embryonic stem cells. The homologous recombination targeting schemes for modifying the *S1pr1* and *Rosa26* loci are shown in (**a**) and (**b**), respectively. For each panel, the WT genes are shown at the top, the targeting vectors are shown in the middle, and the recombined knock-in alleles are shown at the bottom. *Arrb2,* β-arrestin2; PA, polyadenylation sequence; PGK DTA, phosphoglycerate kinase I promoter and A subunit of the diphtheria toxin gene; NeoR, neomycin resistance gene; triangles, loxP sites; E1, exon 1; E2, exon 2; TK, thymidine kinase; CLuc, C-terminal firefly luciferase fragment (amino acids # 394–550); N-Luc, N-terminal firefly luciferase fragment (amino acids # 1–416).

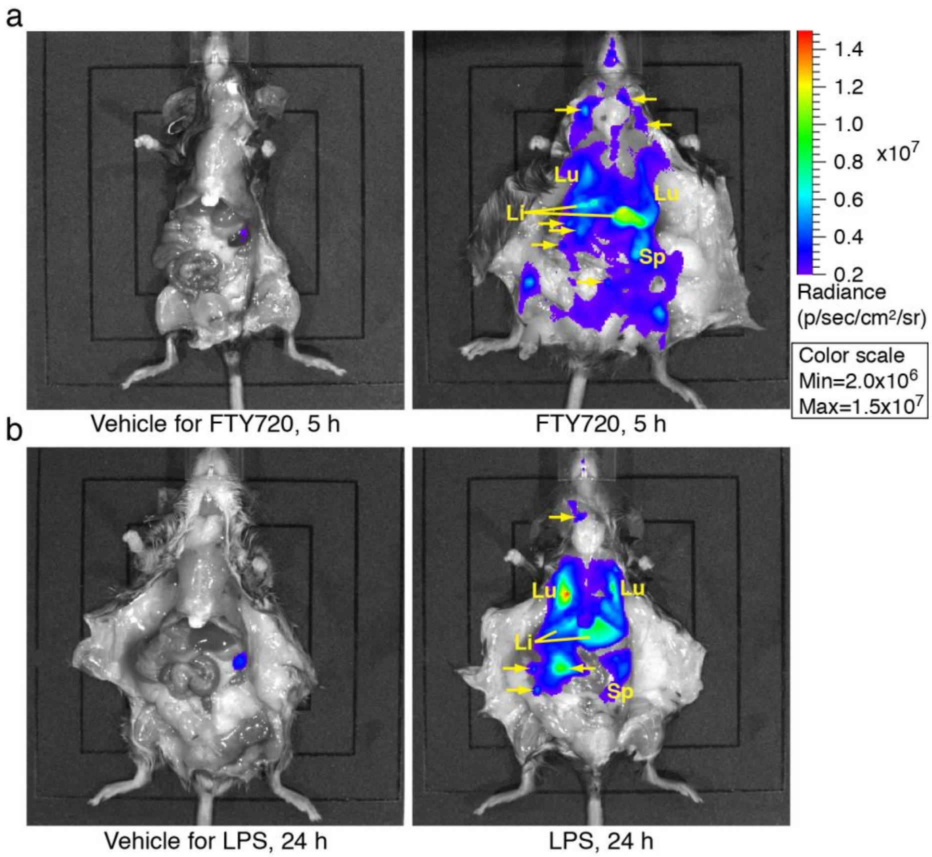

Supplementary Figure 3. $S1P_1$ activation by FTY720 and LPS in internal organs. $S1P_1$ luciferase signaling mice were injected intraperitoneally with FTY720 (**a**) or LPS (**b**) and imaged with organs exposed to identify specific regions with high $S1P_1$ activation. Control mice received vehicle. While under deep anesthesia, the groups of mice (n = 5) were subjected to BLI after the thoracic region and abdominal cavity were opened, 5 h or 24 h after FTY720 and vehicle, or LPS and vehicle, administration, respectively. A representative image from each group of mice is shown. Mice were imaged in the supine position. Intensities are shown in units of photons/sec/cm^2/steradian (p/sec/cm^2/sr). Arrows, lymph node; Li, liver; Sp, spleen; Lu, lung.

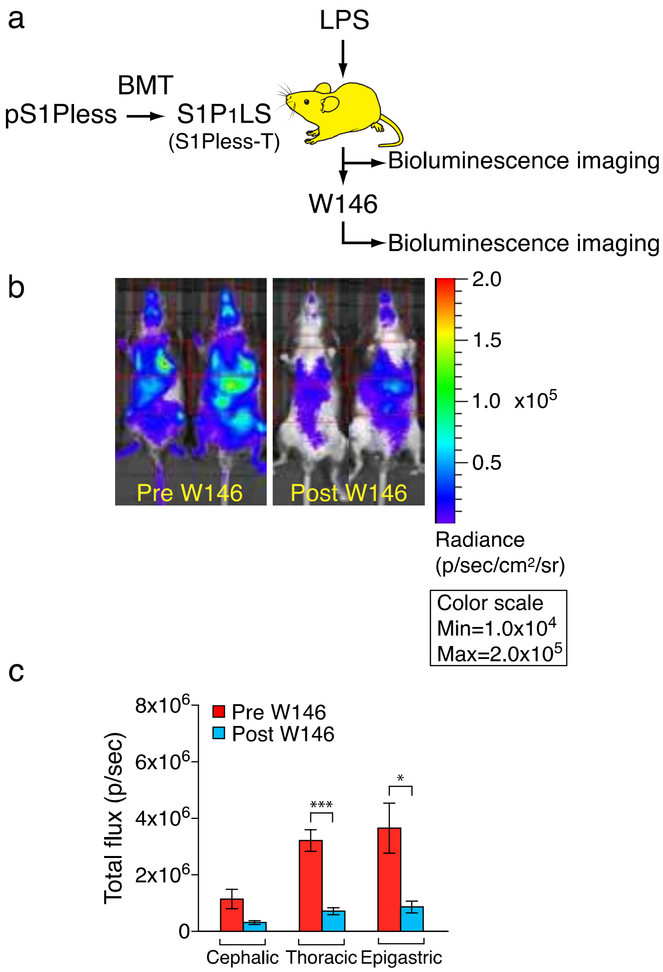

Supplementary Figure 4. Inhibition of $S1P_1$ activation by an $S1P_1$ antagonist during LPS-induced systemic inflammation in live mice transplanted with plasmaS1Pless bone marrow. **a,** Bone-marrow cells from plasma(p)S1Pless mice were transplanted (BMT) into irradiated $S1P_1$ luciferase signaling ($S1P_1LS$) mice. LPS was injected intraperitoneally into the stably transplanted mice 10 weeks later. After 24 h, the mice were intraperitoneally injected with the $S1P_1$ antagonist W146 (10 mg/kg). Mice were subjected to BLI immediately prior to and 0.5 h post-W146 injection. **b,** Representative bioluminescence images of the same $S1P_1$ luciferase signaling mice transplanted with pS1Pless bone marrow immediately prior to and 0.5 h post-W146 injection. Mice were imaged in the supine position. Red open rectangles representing regions of interest (ROI) were positioned around cephalic, thoracic, and epigastric regions. **c,** The

bioluminescence activity was quantified by determining the total flux (photons/sec; p/sec) in each ROI. Data represent the mean ± SEM. n=6 for both groups of mice. *P*-values were determined by two-tailed Student's *t*-test; *, $P \leq 0.05$, ***, $P \leq 0.001$.

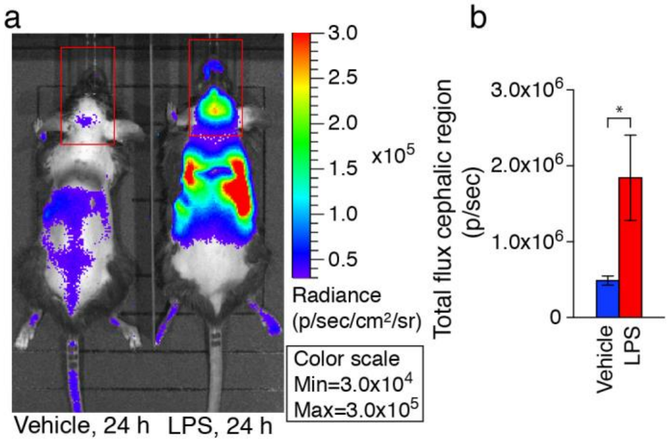

Supplementary Figure 5. S1P$_1$ activation during LPS-induced systemic inflammation in the cephalic region. S1P$_1$ luciferase signaling mice were injected intraperitoneally with vehicle or LPS to induce systemic inflammation, then subjected to BLI. **a,** Representative bioluminescence image of mice 24 h after LPS or vehicle injection. Mice were imaged in the prone position. A red open rectangle representing the ROI was positioned around the cephalic region. **b,** The bioluminescence activity was quantified by determining the total flux (photons/sec; p/sec) in the cephalic ROI. Data represent the mean ± SEM. n=8 for PBS, n=9 for LPS. *P*-values were determined by two-tailed Student *t*-test; *, $P \leq 0.05$.

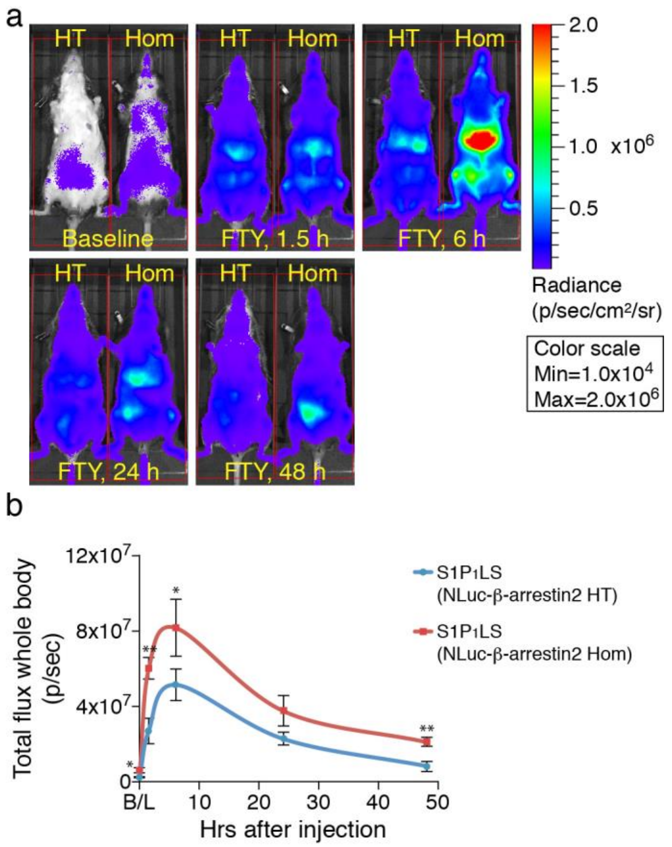

Supplementary Figure 6. $S1P_1$ activation by FTY720 in live mice heterozygous or homozygous for the NLuc–β-arrestin2 allele. Mice were injected with FTY720 (FTY) and subjected to BLI prior to injection (Baseline, B/L) and 1.5, 6, 24, and 48 h after injection. Two genotypes were used: heterozygous for the $S1P_1$-CLuc allele ($S1P_1$-CLuc) and heterozygous for the NLuc–β-arrestin2 allele (HT); and heterozygous for the $S1P_1$-CLuc allele and homozygous for the NLuc–β-arrestin2 allele (Hom). **a,** Representative bioluminescence images of the same mice at specified time points. Mice were imaged in the supine position. A red open rectangle representing the ROI was positioned around the whole body. **b,** The bioluminescence activity was quantified by determining the total flux (photons/sec; p/sec) in the ROI. Data represent the mean ± SEM. n=5 for NLuc–β-arrestin2 heterozygous mice, n=8 for NLuc–β-arrestin2 homozygous mice. *P*-values were determined between NLuc–β-arrestin2 heterozygous and homozygous

groups at each time point by one-way ANOVA followed by Bonferroni's multiple comparisons test; *, $P \leq 0.05$, **, $P \leq 0.01$.
